# A Case of Umbilical Endometriosis: Villar’s Nodule

**DOI:** 10.7759/cureus.926

**Published:** 2016-12-12

**Authors:** Amanda M Krantz, Atman A Dave, Daniel J Margolin

**Affiliations:** 1 School of Medicine, Creighton University; 2 Medical Education, Saint Luke’s Hospital of Kansas City; 3 Surgery, University of Missouri–Kansas City School of Medicine

**Keywords:** endometriosis, villar's nodule

## Abstract

Umbilical endometriosis is a fairly rare clinical entity with unclear pathogenesis. We report the case of a 27-year-old woman who presented with a painful umbilical mass and discharge. Imaging performed was inconclusive, and surgical excision of the site with margins revealed endometriosis on microscopic examination. The incidence of umbilical endometriosis, its pathogenesis, clinical manifestations, workup, and management are discussed.

## Introduction

Endometriosis is a common disease characterized by endometrial tissue located outside of the uterus. Clinically, endometriosis is associated with infertility and pelvic pain. Sites most commonly affected by endometriosis are the pelvic organs, especially the ovaries and fallopian tubes. However, extra-pelvic sites of endometriosis have been described within virtually all organ systems, including the genitourinary tract, bowels, and even the brain [1]. Noninvasive tests including diagnostic imaging or serum markers have not yet been identified that can diagnose endometriosis with accurate sensitivity and specificity. As a result, endometriosis can be difficult to diagnose; definitive diagnosis is performed by visual inspection via laparotomy or laparoscopy [1]. Umbilical endometriosis, also known as Villar’s nodule, is a relatively rare occurrence and is often a result of iatrogenic seeding in surgical scars. Umbilical endometriosis in the absence of any prior abdominal or uterine surgery is an even rarer clinical entity.

## Case presentation

A 27-year-old female, gravida 4 para 3, presented with a persistent umbilical mass from over a year. She had previously undergone three low-transverse cesarean sections and had one miscarriage. She reported constant sharp pain in the mass as well as associated increasing size and worsening discomfort, particularly with her menses. The mass was also reported to have sporadic drainage that ranged from cloudy to clear. Her medical history was remarkable for dyspareunia and intermittent pelvic pain. The patient agreed to participate and was explained the nature and objectives of this study, and informed consent was formally obtained. No reference to the patient's identity was made at any stage during data analysis or in the report.

**Figure 1:**
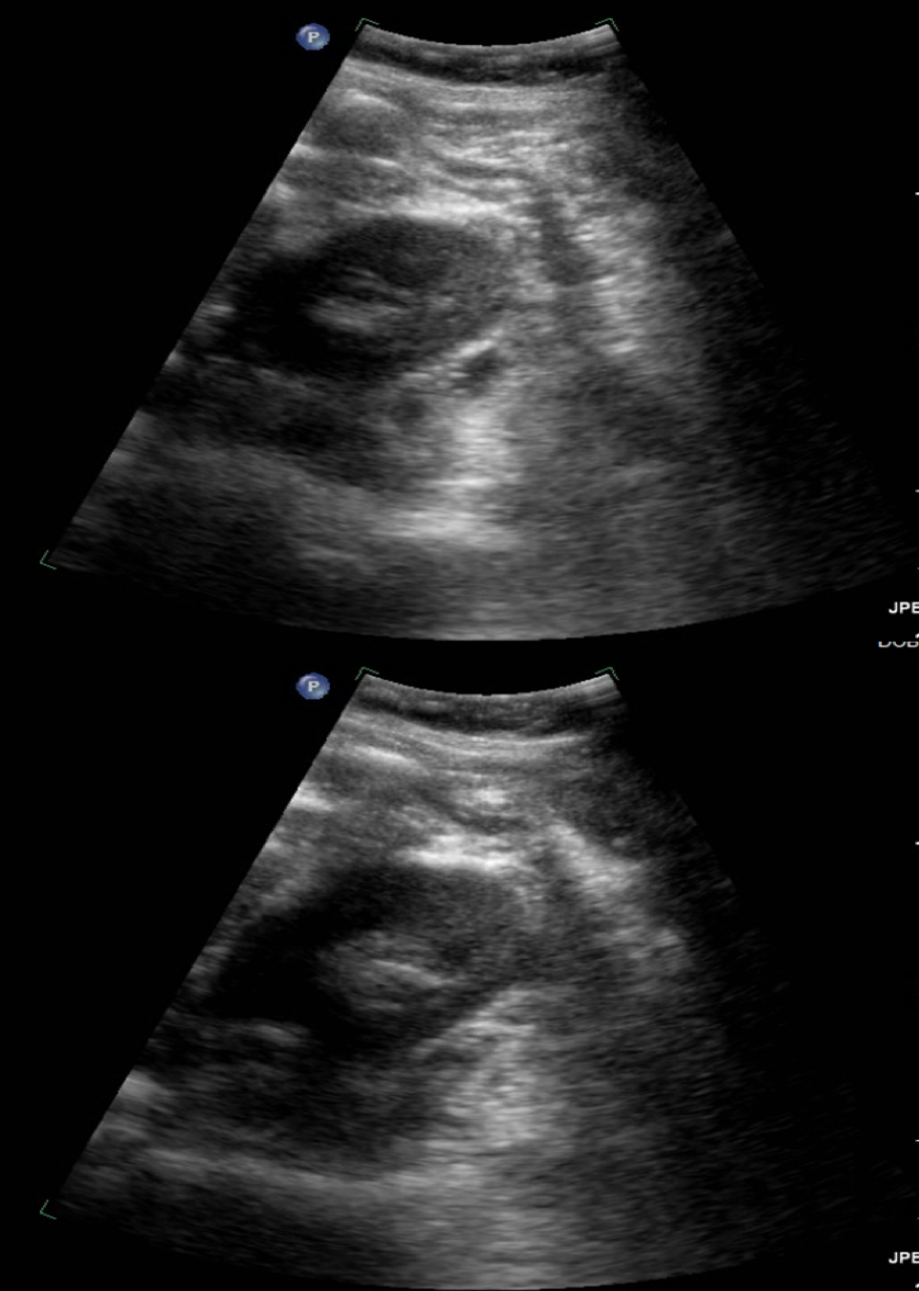
Ultrasonography Findings Ultrasonography findings reflect a heterogeneous lobular complex partially cystic lesion measuring approximately 2.8 x 2.4 x 1.6 cm with a soft-tissue component demonstrating internal vascularity in the subcutaneous soft tissues at the umbilicus.

Physical exam revealed a 2.5 cm firm and indurated subcutaneous mass in the center of her umbilicus, with extreme tenderness to palpation. Ultrasonography revealed an indeterminate solid and cystic lesion in the subcutaneous soft tissues at the umbilicus suggestive of a urachal cyst (Figure 1).

A provisional diagnosis of a urachal sinus tract with a cyst was made. The patient consented to an excision of the umbilical cyst with a diagnostic laparoscopy and plans to close off the urachal sinus tract.

Under general anesthesia, laparoscopic exploration revealed a uterus adherent to the anterior abdominal wall consistent with the patient's history of previous cesarean sections. The area around the umbilicus was examined, and no distinct connection to the bladder was noted. The surrounding tissue around the umbilicus was taken down using a tissue sealer. The umbilicus itself was examined, and no intraabdominal connections were noted. As a result, the laparoscopic approach was concluded, and attention was turned to the umbilicus externally where a circumferential incision was made around the mass. The firm mass was encountered subcutaneously and was circumferentially excised. Because of its adherence to the fascia, a 2 x 2 cm circle of fascia was excised along with the mass. This was sent to pathology for specimen analysis. The fascia was then closed, and the umbilicus was reconstructed. On microscopic examination, histologic sections revealed tissue consistent with endometriosis.

## Discussion

Endometriosis is a frequently encountered clinicopathologic entity with prevalence in fertile women, found to be between 5% and 10% [2]. While the precise pathophysiology of endometriosis is unknown, a widely accepted concept is that the disease develops from a deposition of endometrial cells upon pelvic peritoneum by retrograde menstruation through the fallopian tubes; women with endometriosis have higher volumes of refluxed menstrual blood [2]. Cases of induced endometriosis have also been demonstrated in women with vaginal outlet obstruction [2]. Other circulated theories include the ectopic presence of endometrial stem cells or immune system defects. Investigations into the genetic risk factors for endometriosis revealed that alterations to p53, PTEN, Cytochrome P450 1A1, and Peroxisome proliferator-activated receptor γ2 Pro-12-Ala have been implicated in endometriosis [2]. Prologed radiation exposure as well as increased estrogen exposure have also been implicated [2].

Ectopy of endometrial tissue in the umbilicus represents between 0.5% and 1.0% of extrapelvic endometriosis [3]. Umbilical endometriosis can be differentiated into primary and secondary types, with the former defined by a distinct lack of previous gynecological surgery or cesarean section. The pathogenesis of both types of umbilical endometrioses is not fully elucidated.

Primary umbilical endometriosis is thought to arise from lymphatic or hematogenous spread, namely along the lymphatic channels that join the peritoneum along obliterated umbilical vessels or by metaplasia of remnant urachal tissue into endometrial tissue through inflammatory changes.

In our case, the patient’s history of low transverse caesarean sections was more consistent with secondary umbilical endometriosis. The description of this term comes from an iatrogenic seeding of endometrial cells, which most commonly has been noted to occur in low transverse abdominal scars after cesarean section in 1% of patients [4].

The dissemination of endometrial implants to the umbilical region and other areas may occur in a surgical setting where direct exposure of endometrial tissue occurs to tissue tracts and blood vessels exposed by mechanical disruption. The fibrin-rich surfaces of incisions provide viable sites for deposition of angiogenic endometrial implants. Furthermore, it has been hypothesized that the umbilicus may act as a physiological scar and seeding site, contributing to the etiology of secondary umbilical endometriosis [5].

Presentation of umbilical endometriosis often involves an umbilical mass of variable size, up to 6 cm, as well as localized symptoms such as cyclic pain (81.5%), bleeding (49.2%), and swelling (90.9%) [6]. Patients often have a lengthy course before presentation, with an average duration of symptoms prior to presentation and evaluation over a year. The most common demographic for umbilical endometriosis is premenopausal ovarian-steroid-dependent women; a mean age in one study was found to be 37.7 years old while the youngest was noted to be 23 years old [6].

A workup, after a thorough history is taken from the patient, often involves imaging, particularly ultrasonography, to assess the echogenicity and vascular involvement. Magnetic resonance imaging can also be helpful, although no imaging modality is truly diagnostic [5]. Notably, fine needle aspiration has been shown to be inconclusive in the majority of cases [7].

Although the management of umbilical endometriosis is not standardized, the approach to definitive treatment is generally surgical. Endometriomas may generally respond to aggressive medical therapy but often require prolonged therapy for symptomatic control, which is rarely curative [6]. The ability to excise the endometriotic tissue is dependent on the local extent of the growth and exact implantation site. Total excision of the umbilicus with an underlying repair and reconstruction is one option, while local resection of the endometriotic tissue may also be performed to spare the umbilicus [8]. In both approaches, a margin of tissue around the endometriotic site is recommended to prevent local recurrence, and some medical experts recommend full umbilical excision regardless of the size or local involvement [9].

This case is notable for the younger age of the patient in the appropriate clinical setting of cyclical pain and discharge with the umbilical mass after three low-transverse cesarean sections.

## Conclusions

Umbilical endometriosis should be suspected in the clinical setting of an umbilical mass, especially in the premenopausal female with a surgical history. This case represents endometriosis in a younger patient, with the classical signs and symptoms of umbilical endometriosis, as well as a convincing predisposing past medical history. Surgical management was undertaken and is generally the favored approach for definitive treatment.
